# Aberrant Localization of FUS and TDP43 Is Associated with Misfolding of SOD1 in Amyotrophic Lateral Sclerosis

**DOI:** 10.1371/journal.pone.0035050

**Published:** 2012-04-06

**Authors:** Edward Pokrishevsky, Leslie I. Grad, Masoud Yousefi, Jing Wang, Ian R. Mackenzie, Neil R. Cashman

**Affiliations:** 1 Brain Research Centre, University of British Columbia, Vancouver, Canada; 2 Department of Pathology and Laboratory Medicine, University of British Columbia, Vancouver, Canada; National Institutes of Health, United States of America

## Abstract

**Background:**

Amyotrophic lateral sclerosis (ALS) is incurable and characterized by progressive paralysis of the muscles of the limbs, speech and swallowing, and respiration due to the progressive degeneration of voluntary motor neurons. Clinically indistinguishable ALS can be caused by genetic mutations of Cu/Zn superoxide dismutase (SOD1), TAR-DNA binding protein 43 (TDP43), or fused in sarcoma/translocated in liposarcoma (FUS/TLS), or can occur in the absence of known mutation as sporadic disease. In this study, we tested the hypothesis that FUS/TLS and TDP43 gain new pathogenic functions upon aberrant accumulation in the cytosol that directly or indirectly include misfolding of SOD1.

**Methodology/Principal Findings:**

Patient spinal cord necropsy immunohistochemistry with SOD1 misfolding-specific antibodies revealed misfolded SOD1 in perikarya and motor axons of SOD1-familial ALS (SOD1-FALS), and in motor axons of R521C-FUS FALS and sporadic ALS (SALS) with cytoplasmic TDP43 inclusions. SOD1 misfolding and oxidation was also detected using immunocytochemistry and quantitative immunoprecipitation of human neuroblastoma SH-SY5Y cells as well as cultured murine spinal neural cells transgenic for human wtSOD1, which were transiently transfected with human cytosolic mutant FUS or TDP43, or wtTDP43.

**Conclusion/Significance:**

We conclude that cytosolic mislocalization of FUS or TDP43 *in vitro* and ALS *in vivo* may kindle wtSOD1 misfolding in non-SOD1 FALS and SALS. The lack of immunohistochemical compartmental co-localization of misfolded SOD1 with cytosolic TDP43 or FUS suggests an indirect induction of SOD1 misfolding followed by propagation through template directed misfolding beyond its site of inception. The identification of a final common pathway in the molecular pathogenesis of ALS provides a treatment target for this devastating disease.

## Introduction

ALS is characterized by progressive paralysis of the muscles of the limbs, speech, swallowing and respiration, due to the progressive degeneration of innervating motor neurons [1]. Certain proteins, mutant and wild-type alike, have been identified to mislocalize or accumulate as microscopically identifiable misfolded aggregates that may participate in ALS pathogenesis [2]–[4]. Misfolded aggregated proteins and peptides are also thought to participate in prion diseases, such as Creutzfeldt-Jakob, kuru and fatal familial insomnia diseases [5]–[6] and other neurodegenerative diseases, including Alzheimer’s disease, Parkinson’s disease, frontotemporal dementia (FTLD) and Huntington’s disease [7]–[8]. In FALS, mutations in the gene encoding the Cu/Zn superoxide dismutase (SOD1), a ubiquitously-expressed free-radical defense enzyme, are associated with misfolding of this normally stable homodimeric protein [9]–[10]_ENREF_7. Recent studies have also detected misfolded SOD1 in sporadic forms of the disease in which SOD1 mutation is excluded [2], [11], suggesting that non-native conformers of SOD1 may participate in a common pathological mechanism shared by all types of ALS. In addition to SOD1, heritable mutation of two other genes are implicated in FALS, and associated with protein mislocalization and aggregation: the RNA-processing proteins fused in sarcoma (FUS), originally named translocated in liposarcoma (TLS), and TAR-DNA binding protein 43 (TDP43) [12]–[14]_ENREF_10_ENREF_10. FUS/TLS and TDP43 are primarily nuclear proteins that participate in common heteromultimeric complexes [15] involved in RNA transcription, translation, splicing, nucleo-cytoplasmic shuttling, transport for local translation, and stress granule formation [16]. These proteins belong to the family of heterogeneous nuclear ribonucleoproteins, which are thought to interact with other proteins through glycine-rich domains [17]. Under pathological circumstances, the predominantly nuclear FUS/TLS and TDP43 are redistributed to the cytosol wherein they participate in abnormal protein-protein association in this aberrant locale [18]–[19]. TDP43 can accumulate in the neuronal cytosol in other neurodegenerative diseases (e.g. Alzheimer’s disease), in which it may be a marker of cell stressors [16]. Moreover, naturally occurring mutations in FUS/TLS (hereafter referred to as FUS) and TDP43 lead to formation of cytoplasmic aggregates that are associated with a limited number of heritable syndromes, including ALS [16].

In this study, we tested the hypothesis that FUS and TDP43 gain new pathogenic functions upon accumulation in the cytosol that directly or indirectly includes misfolding of SOD1. We detected axonal misfolded SOD1 in a spinal cord from a FUS-FALS patient, and from SALS patients with TDP43 pathology. We also used HA-tagged FUS and TDP43 to demonstrate that expression of cytosolic mutant FUS/TLS or TDP43 is associated with misfolding of endogenous human wild-type SOD1, as detected by SOD1 misfolding-specific antibodies directed against a disordered electrostatic loop [20]–[21], or monomerized and oxidized SOD1 [22]. Furthermore, over-expression of wild-type TDP43, but not wild-type FUS, is also associated with cytosolic localization and SOD1 misfolding.

## Results

### SOD1 Misfolding in SOD1-FALS, FUS-FALS and SALS with TDP43 Pathology

**Figure 1:**
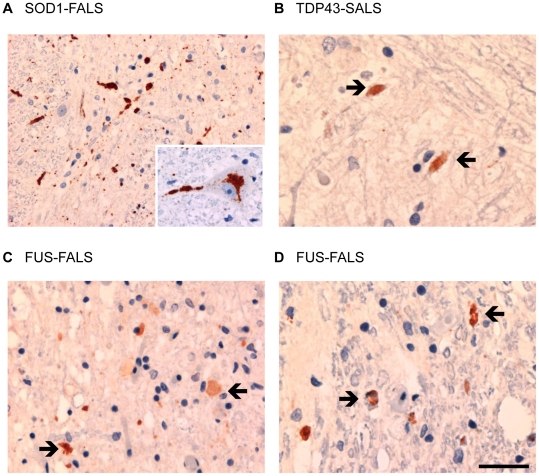
Misfolded SOD1 immunohistochemistry in ventral grey matter and corticospinal tracts of cervical spinal cord of ALS patients. (**A**) Cases of FALS with SOD1 mutations show extensive accumulation of misfolded SOD1 in swollen and normal sized axons as well as in the perikarya of some lower motor neurons (inset). (**B**) Cases of SALS with TDP43 pathology had misfolded SOD1 accumulation only in small numbers of normal sized axons (arrows). (**C**, **D**) In the case of FALS with FUS mutation misfolded SOD1 accumulated in some swollen axons in the ventral grey matter (**C**, arrows) and a moderate number of normal sized axons in corticospinal tract (**D**, arrows). Scale bar, 60 µm (**A**, **C**); 30 µm (**B**, **D**).

Immunohistochemistry was performed on formalin-fixed paraffin-embedded cervical spinal cord sections from FALS and SALS patients (Figure 1) using the mouse monoclonal disease specific epitope antibodies (mAb DSE) 10E11C11 or 3H1, both generated against a linear peptide corresponding to a structurally disrupted SOD1 electrostatic loop [9], [20] detectable only when the protein is misfolded in disease [20]–[21]. Cases of FALS with SOD1 mutations (N = 5) had the most extensive accumulation of misfolded SOD1 in neuronal cytoplasmic inclusions, dystrophic swollen axons and normal sized axons (Figure 1A). Cases of SALS with TDP43 pathology (N = 3) had misfolded SOD1 in only a small number of normal sized axons as a consistent feature (Figure 1B). A case of FALS with the R521C-FUS mutation, with a known display of substantial cytoplasmic FUS localization and negative for TDP43 pathology [13], [23], revealed an intermediate amount of misfolded SOD1 accumulation in normal sized axons in the ventral grey matter (Figure 1C) and occasional swollen axons in the corticospinal tract (Figure 1D), but not within neuronal perikarya. We further used 10E11C11 to demonstrate immunoreactivity for misfolded SOD1 in non-SOD1 FALS (N = 3) and in SALS (N = 20), but not in normal controls (N = 5) (Table 1). All the available demographic and clinical information for these samples are presented in Table S1.

**Table 1:** Detection of misfolded SOD1 in various structures and regions by immunohistochemistry.

Structure/Region	SOD1-FALS	non-SOD1 FALS	non-SOD1 SALS	Control
Neuronal cytoplasmic inclusions	++	−	−	−
Axonal swellings	++	−	−	−
Axons	+++	++	+	−
Corticospinal tract	+++	++	++	−
Other tracts	+	+	+	−
Motor Roots	++	+	+	−

Relative abundance of DSE antibody (10E11C11) staining in various regions and structures of patient spinal cord sections (N = 5 normal control; N = 5 SOD1-FALS; N = 3 non-SOD1 FALS including R521C-FUS; N = 20 non-SOD1 SALS). Neuronal cytoplasmic inclusions, axonal swellings and axons are sub-structures within the ventral grey matter. Positive staining in corticospinal tract, other tracts and motor roots was in axons. –, no staining; +, some staining; ++, moderate staining; +++, abundant staining.

### Cytosolic FUS or TDP43 and SOD1 Misfolding in Neural Cells

**Figure 2:**
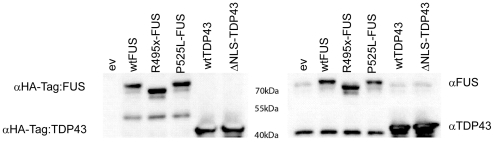
Expression of transfected wild-type and mutant FUS and TDP43 in SH-SY5Y cells. The immunoblot on the left was probed with antibody specific to HA-tag, detecting only the exogenously expressed proteins in these samples. The empty vector (ev; pCINeo) control does not display detectable immunoreactivity with the HA-tag antibody, while both exogenous FUS and TDP43 are detected as 43 kDa and 72 kDa bands, respectively. Top and bottom portions of the immunoblot on the right were probed using FUS and TDP43 specific antibodies, respectively, detecting both the endogenous and exogenous proteins. Endogenous FUS and TDP43 are detected in all samples; however, stronger signals, corresponding to the over-expressed protein, are detected in the transfected samples.

In order to examine the effects of aberrantly localized FUS and TDP43 on the folding of human wild-type SOD1 (wtSOD1), we transfected human neuroblastoma SH-SY5Y cells with expression constructs of wild-type and mutant FUS and TDP43, including the naturally occurring FUS cytosol-localizing truncation mutation R495x [24] and the missense mutant P525L [12], [18], as well as the experimentally-engineered cytosol-localizing triple missense tandem TDP43 mutation (K82A, R83A, K84A) in the nuclear localization signal (NLS; mutant named ΔNLS-TDP43) [25]. Figure 2 confirms the expression of all transfected constructs by showing that probing the immunoblot with HA-Tag antibody detects only the exogenous FUS and TDP43 protein, whereas probing with FUS- and TDP43- specific antibodies leads to detection of both the endogenous and exogenous proteins. Endogenous FUS is seen as a faint band in all the SH-SY5Y cell lysate samples, whereas the darker bands include the exogenous FUS detectable only in wt, R495x, or P525L-FUS expressing cells, with the truncation mutant being smaller in mass. Endogenous TDP43 is also detected in all SH-SY5Y cell lysate samples, with wt and dNLS-TDP43 transfected cells showing significantly more immunoreactivity.

**Figure 3:**
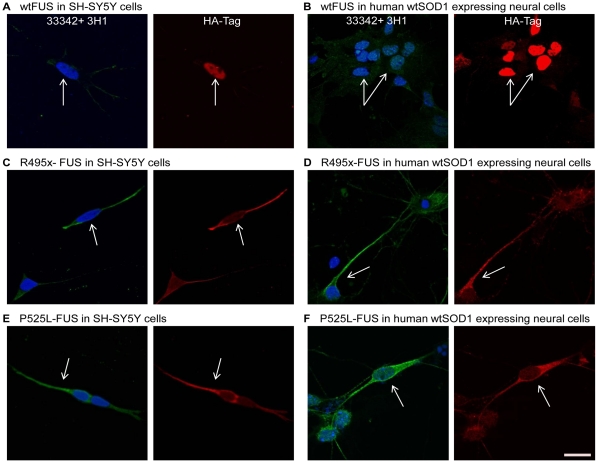
Transfection of mutant FUS is associated with SOD1 misfolding by immunocytochemistry. Human neuroblastoma SH-SY5Y cells and primary neural cultures expressing human wtSOD1 were stained for HA-Tag (red), misfolded SOD1 (green), and Hoechst33342 nuclear counterstain (blue). (**A**, **B**) Human wild-type FUS localizes in the nucleus and no misfolded SOD1 is detected. (**C**–**F**) Both of the truncated variant, R495x-FUS (**B**, **C**), and point mutation variant, P525L-FUS (**E**, **F**), localize in the cytosol and are associated with misfolding of SOD1 in the same cells, as detected by the immunocytochemistry with the 3H1 SOD1 misfolding-specific mAb. Exogenous FUS was detected using the N-terminal HA-tag. Arrows point to transfected cells. Scale bar, 20µm.

**Figure 4:**
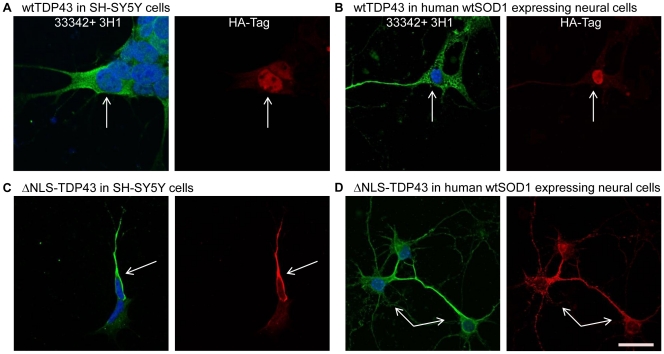
SOD1 misfolding in wild-type and ΔNLS-TDP43-transfected SH-SY5Y cells and human wtSOD1 expressing primary neural cells. SH-SY5Y cells and primary neural cells stained against HA-tag (transfected TDP43; red), misfolded SOD1 (green), and Hoechst33342 (blue) nuclear counterstain. Human wtTDP43 predominantly localizes in the nucleus (**A**, **B**) while the mutant ΔNLS-TDP43 localizes in the cytosol (**C**, **D**). Both variants of TDP43 are associated with misfolding of the endogenous SOD1 in the same cells, as detected by 3H1 immunoreactivity. Arrows point to transfected cells. Scale bar, 20µm.

We have found that murine wtSOD1 is not efficiently misfolded by human mutant SOD1 [26]. To recapitulate as best as possible in primary neural cells the potential effect of FUS and TDP43 on the folding of human wtSOD1, E12–14 dissociated spinal cord cultures were prepared from transgenic mice expressing human wtSOD1. We did not select for neurons exclusively as the expert consensus is that ALS is a non-cell autonomous disease, that is both neurons and non-neuronal cells participate in the pathogenesis [27]. In the human SH-SY5Y cell line, endogenously expressed or transfection-driven wtFUS and wtTDP43 localize to the nucleus by immunocytochemistry (Figure 3A, 3B; Figure 4A, 4B; Figure S1), although a minor fraction of over-expressed wtTDP43 appeared in the cytosol. In SH-SY5Y cells and in primary neural cultures, expression of extranuclear FUS mutants R495x and P525L, or the TDP43 mutant, ΔNLS-TDP43, was associated with endogenous SOD1 misfolding in the cytosol of the same cells as detected by reactivity with the SOD1 DSE mAb 3H1 (Figure 3 and Figure 4). Five to seven percent of SH-SY5Y cells and ∼ 1% of primary neural cells were successfully transfected and displayed cytosolic localization of transgenic mutant FUS and TDP43, of which the majority (range 50–75%) also exhibited 3H1 immunoreactivity. Transfected SH-SY5Y cells or human wtSOD1-expressing primary neural cells demonstrate the presence of 3H1-immunoreactive misfolded SOD1 in FUS- (Figure 3C–F) or TDP43- (Figure 4A–D) transfected cells, but not in non-transfected cells in the same fields of view. Differences in SOD1 misfolding were observed between wtFUS and wtTDP43 transfection: exogenous wtFUS was confined to the nucleus in SH-SY5Y and primary neural cells without detectable 3H1 immunoreactivity in these cells (Figure 3A, 3B), whereas exogenous wtTDP43 was predominantly distributed in the nuclear compartment with trace cytoplasmic immunoreactivity, and was strongly associated with SOD1 misfolding in the same cells (Figure 4A, 4B).

### Quantification of FUS- or TDP43-associated SOD1 Misfolding

**Figure 5:**
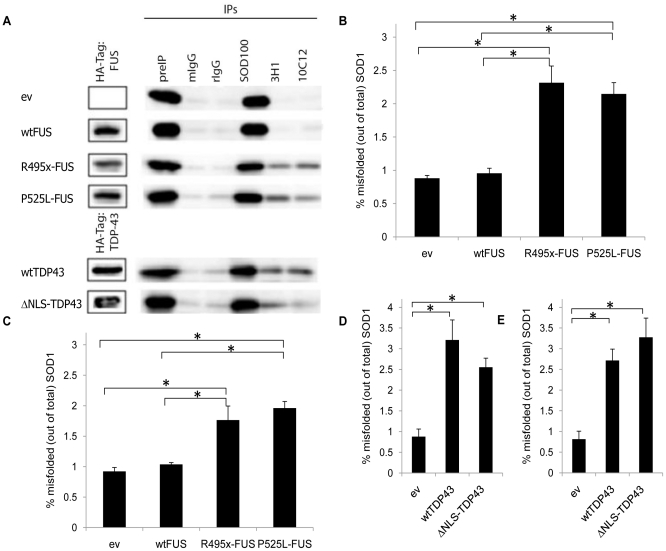
Quantitative immunoprecipitation of misfolded human SOD1 in SH-SY5Y cells transfected with wild-type or mutant FUS and TDP43. (**A**) Representative immunoblots of immunoprecipitations. SOD1 proteins from transfected (48 h) and untransfected SH-SY5Y cell lysates were precipitated using pan-SOD1 antibody, SOD100 (rabbit polyclonal), and SOD1 misfolding-specific mouse monoclonal antibodies, 3H1 and 10C12. rIgG was used as isotype control for SOD100, and mIgG2a was used as isotype control for the DSE antibodies. Blots were probed with pan-SOD1 antibody. Framed bands show FUS, and TDP43 transfection efficiency of the indicated construct, as was detected by probing with antibody to HA-tag. (**B**–**E**) Show percentage of immunoprecipitable misfolded SOD1 (out of the total precipitable SOD1) using 3H1 (**B**, **D**) and 10C12 (**C**, **E**) from lysates of transfected SH-SY5Y cell cultures. Appreciable differences are indicated (*, *p*<0.01). N = 5 for each of wtFUS, R495x- and P525L-FUS. N = 6 for each of ev, wtTDP43 and ΔNLS-TDP43. Error bars represent s.e.m.

Quantitative immunoprecipitation of non-denatured lysates from transfected SH-SY5Y cells was performed with magnetic beads coupled with DSE 3H1 mAb and the SOD1 misfolding/oxidation-specific DSE 10C12 mAb, generated against a cysteic acid-substituted SOD1 exposed dimer interface (SEDI) peptide [22], [26]_ENREF_25 (Figure 5A). Specific immunoprecipitation of conformation-specific 3H1 and 10C12 mAbs was controlled by irrelevant mouse IgG mAb. Transfection of cells with the pCINeo empty vector (ev) lacking coding DNA provided an additional negative control for cell stressors inherent in the protocol. SOD1 misfolding specific 3H1 and 10C12 mAbs immunoprecipitated appreciable levels of misfolded and/or oxidized endogenous SOD1 in cultured cells transfected with cytosolic mutants R495x- and P525L- FUS compared to ev (Figure 5B–C; 3H1: *p*<0.009, *p*<0.002 and 10C12: *p*<0.006, *p*<0.001, respectively). No misfolded SOD1 was detected in cells transfected with wtFUS (3H1: *p*>0.5 and 10C12: *p*>0.7; compared to ev). Lysates of SH-SY5Y cells transfected with wt- or ΔNLS- TDP43 presented misfolded SOD1 (Figure 5D–E; 3H1: *p*<0.002, *p*<0.001 and 10C12: *p*<0.001, *p*<0.001, respectively). Considering that only 5% of the SH-SY5Y cells were successfully transfected, the immunoprecipitation data suggests that nearly 20–50% of the total cellular SOD1 is misfolded in mutant FUS and TDP43 expressing cells. No misfolded SOD1 was detected using 3H1 and 10C12 mAb when SH-SY5Y cells were transfected with green fluorescence protein (GFP, Figure S2), consistent with the specific effect of FUS and TDP43 cytosolic accumulation on SOD1. The immunoprecipitation studies are in agreement with the immunocytochemical studies, in that cytosolic localization of mutant FUS and TDP43, and wtTDP43, but not wtFUS, is associated with SOD1 misfolding.

## Discussion

Previous studies suggest that a neurotoxic gain of function is incurred by pathological isoforms of FUS and TDP43 that accumulate in the cytosol of afflicted cells [4], [15]. Here we report that motor axons from R521C FUS-FALS and wtTDP43-SALS patient spinal cords are immunoreactive for misfolded SOD1 by immunohistochemistry with SOD1 misfolding-specific mAbs. Using these same antibodies we found that transfection-driven expression of cytosolic mutants of FUS or TDP43 *in vitro* is associated with SOD1 misfolding by immunocytochemistry and immunoprecipitation. Motor axonal misfolded SOD1 was also observed by immunohistochemistry in SALS, in which extranuclear non-mutant TDP43 is ubiquitinated and accumulates in a proteolytically cleaved and detergent insoluble form [14], [23], [28], and in neural cells in which wtTDP43 is over-expressed. Commensurate with these findings, motor neuron disease is induced by transgenic over-expression in rodents of human mutant FUS or TDP43 [29], and by expression of wtTDP43 [30]–[32] but not wtFUS [29]. Cytosolic accumulation of wtFUS aggregates is not observed in SALS [13], and we did not detect SOD1 misfolding in neural cells over-expressing wtFUS. Given the concordance between the neuropathological immunohistochemistry, and the immunocytochemistry and immunoprecipitation of cultured cells, we conclude that cytoplasmic accumulation of FUS and/or TDP43 is associated with misfolding of human wtSOD1, both in *vivo* and *in vitro*.

Is extranuclear accumulation of FUS and TDP43 a cause or consequence of SOD1 misfolding, and if causal is the interaction direct or indirect? Consistent with our studies in cell culture and in human FUS-FALS, a *causal* association is tenable between SOD1 misfolding and cytosolic accumulation of mutant FUS, or mutant and wtTDP43. However, accumulation of non-mutated TDP43 (but not wtFUS) in SALS spinal neuronal cytosol may also be, at least partially, a *consequence* of cell stress, as the pathology also appears in diverse diseases including Alzheimer’s disease and frontotemporal lobar dementia [33]. Recent work has shown that TDP43 aggregation and neurotoxic cleavage may be triggered by free radical stress secondary to mitochondrial dysfunction [34]–[35], a concomitant of cellular mutant SOD1 expression [20]–[21]. Immunohistochemical differences in the distribution and abundance of misfolded SOD1 in FUS-FALS and TDP43-SALS are potentially consistent with the notion of different mechanisms of SOD1 misfolding.

Concerning the question of direct or indirect interaction of cytosolic FUS and/or TDP43 with SOD1, we believe that the lack of immunohistochemical co-distribution of misfolded SOD1 (in motor axons) and FUS or TDP43 (in perikarya) in spinal motor neurons from FUS-FALS patients, as well as from SALS patients with TDP43 deposits [23], suggests that an indirect mechanism of SOD1 misfolding *in vivo*. Indirect mechanisms for FUS- or TDP43-induced misfolding of SOD1 include chaperone titration, for example TDP43 and SOD1-associated heat shock proteins HSP-B8 [36] and HSP70 [37]. Furthermore, FUS and TDP43 participate in a complex that modulates expression of histone deacetylase (HDAC) 6, implicated in clearance of misfolded proteins through the autophagy pathway [15], [38]. Both cytosolic mutant FUS and TDP43 have previously been suggested to sequester endogenous FUS [13], [39] and TDP43 [25], therefore depleting the nucleus of the respective protein.

We here report that SOD1 misfolding is observed with cytosolic accumulation of mutant FUS or TDP43 in FUS-FALS and TDP43-FALS, respectively, as well as of wtTDP43 in SALS. However, SOD1-FALS, wherein motor axons display prominent inclusions of misfolded SOD1, is not associated with cytosolic accumulation of FUS or TDP43 [16]. We propose that SOD1 misfolding could constitute a critical molecular event in the unified pathogenesis of FALS and SALS, distal to FUS or TDP43 cytosolic accumulation. Given that misfolded mutant and wtSOD1 can template the misfolding of natively folded wtSOD1 and propagate from cell to cell *in vitro* [9], [40], an attractive speculation is that stochastic induction of a competent template by mutant SOD1, FUS, or TDP43 could trigger the propagation of SOD1 misfolding in FALS and SALS. Such propagation of SOD1 misfolding by an SOD1 template is also consistent with the lack of congruent distribution in ALS spinal neurons. Misfolded SOD1 acquires a toxic gain of function, which may include generation of reactive oxygen and nitrogen species by direct enzymatic activity [41] and by induction of mitochondrial dysfunction [21]. In SALS, cytoplasmic accumulation of wtTDP43, as a local response to injury or cell stress, may also induce a productive template of misfolded SOD1, or may be a toxic consequence of SOD1 misfolding. The confirmation of a final common molecular pathway in ALS, mediated by the propagated misfolding of SOD1, would have important implications for the effective treatment of this devastating disease.

## Materials and Methods

### Cloning and Mutagenesis

FUS and TDP43 plasmids (Plasmid #21827, AddGene, Cambridge, MA; MHS1010-98051213, Open Biosystems, Huntsville, AL), were amplified and modified using PCR-based site-directed mutagenesis. To avoid steric hindrance effects due to large tagging probes, the small hemagglutinin (HA)-tag, derived from amino acids 98–106 of human influenza hemagglutinin [42], was used to label the amino-termini of our wild-type and mutant constructs so as to distinguish from endogenous proteins. HA-tagging has been successfully used in other studies for detection of exogenous FUS [13], [18] and TDP43 [15], [43]. Amino terminal HA-tag was fused to FUS and TDP43 using: 5′-CCGCTCGAGGCCACCATGTACCCATACGATGTTCCAGATTACGCTGCCTCAAACGATTATACC and 5′-CCGCTCGAGGCCACCATGTACCCATACGATGTTCCAGATTACGCTTCTGAATATATTCGG, respectively. Truncation R495x- and point P525L-mutations were created using the following reverse oligonucleotide primers: 5′- CTAGTCTAGATTAGAAGCCTCCACGGTC and 5′-CTAGTCTAGATTAATACAGCCTCTCCCTGCG, respectively. For the generation of missense tandem mutations in TDP43 (K82A, R83A, and K84A), oligonucleotide primers 5′-CCAAAAGATAACGCAGCAGCAATGGATGAGACAGATGC, and 5′-GCATCTGTCTCATCCATTGCTGCTGCGTTATCTTTTGG were used.

### Tissue Culture and DNA Transfections

We cultured human neuroblastoma SH-SY5Y cells (ATCC, Manassas, VA) in Dulbecco’s Modified Eagle Medium (DMEM) supplemented with 10% FBS, 10 U/ml penicillin, 10 U/ml streptomycin and 2 mM L-glutamine. Cells were transiently transfected using the low cytotoxicity Lipofectamine LTX (Invitrogen, Carlsbad, CA), according to manufacturer’s instructions. For immunofluorescence and immunoprecipitation experiments, cells were cultured in 24-well and 10 cm tissue culture-treated plates, respectively.

### Mouse Primary Spinal Cord Culture

Experiments involving animals were conducted according to the Canadian Council on Animal Care guidelines and have been approved by the Animal Care Committee of the University of British Columbia (Approval ID: A10-0212). Pregnant C57 BL/6 female mice (Strain: B6SJL-Tg(SOD1)2Gur/J, Stock:002297; Jackson Laboratories, Bar Harbor, ME) were sacrificed according to the guidelines of the Institutional Animal Care and Use Committee (IACUC). Primary cerebral cortical cultures were prepared from 12–14 day fetal mice using minor modification of an established protocol [44]. Following embryo genotyping, cervical, thoracic and lumbar- regions of the spinal cord were dissected out in Ca^2+^/Mg^2+^-free Hanks Balanced Salts (GIBCO BRL, Grand Island, NY). Meninges were removed and the tissue was transferred to 0.25% trypsin (GIBCO BRL, Grand Island, NY) and digested at 37°C for 15 min. Tissue was then resuspended in DMEM (GIBCO BRL, Grand Island, NY) plus 10% fetal bovine serum (GIBCO BRL, Grand Island, NY) and triturated 4–6 times through a fire-polished tip. The supernatant was centrifuged at 200 × g for 45 sec. Pelleted neural cells were resuspended in Neurobasal media (GIBCO BRL, Grand Island, NY), B27 (GIBCO BRL, Grand Island, NY), 2 mM L-glutamine (Sigma, Saint Louis, MO) and seeded at a density of 2 × 10^5^ cells/well onto poly-D-lysine (Sigma, Saint Louis, MO) coated #1.5 coverslips in 24-well plates. Cultures were maintained in serum-free Neurobasal-B27 medium, and one-half of medium was replaced on day 3 or 4 with equal volume of fresh medium. Cells were transfected at 5 DIV using Lipofectamine LTX with Plus reagent (Invitrogen, Carlsbad, CA).

### Embryo Genotyping

To mimic the scenario of association between human wtSOD1, and FUS and TDP43 in primary neurons, neural cultures were prepared from human wtSOD1 expressing C57 BL/6 Tg mouse embryos. Tissue was collected in 200 µl of lysis buffer (0.45% Nonidet P−40, 0.45% Tween 20, 50 mM KCl, 10 mM Tris-HCl in water, pH 8.8) with 2 µl of Proteinase K (Invitrogen, Carlsbad, CA), and incubated at 60°C with constant shaking for 2 h. The sample was then incubated at 96°C for 10 min to inactivate the proteinase K. Immediately upon inactivation, the PCR mixture was prepared using 2 µl of lysis buffer containing DNA for genotyping. Two sets of primers were used for the PCR reaction: 5′-CATCAGCCCTAATCCATCTGA and 5′-CGCGACTAACAATCAAAGTGA (hwtSOD1 specific primers); 5′-TGGACAGGACTGGACCT CTGCTTTCCTAGA and 5′-TAGAGCTTTGCCACATCACAGGTCATTCAG (internal mouse control).

### Immunofluorescence

In order to detect specifically misfolded SOD1, we used the disease specific epitope (DSE) monoclonal antibody 3H1 [9], [20], [26]. SH-SY5Y cells were placed on glass cover-slips (#1.5) in a 24-well plate one day prior to transfection. 48 h post transfection, cells were washed once with ice-cold PBS and fixed in 4% paraformaldehyde (in PBS, pH 7.4) for 15 min at room temperature. Fixed cells were then permeabilized twice for 5 min in PBST (0.3% Triton X-100 in PBS) and incubated with 2 µg/ml mouse monoclonal 3H1, as well as one of 10 µg/ml rabbit TDP43 specific (ProteinTech Group Inc., Chicago, IL), FUS specific (Abcam, Cambridge, MA) or HA-tag specific (Thermo Scientific, Rockford, IL) polyclonal antibody, diluted in PBST with 2% normal goat serum, for 1 h at room temperature. Cells were then washed twice in PBST and incubated with appropriate secondary antibody conjugated to Alexa Fluor 488 (green) or Alexa Fluor 647 (red) dyes (Invitrogen, Carlsbad, CA; both diluted 1∶1,000) for 1 h at room temperature in the dark. For DNA counter-staining, bisBenzimide H33342 trihydrochloride (Hoechst 33342) was used at 2 µg/ml (in PBST). Hoechst33342-stained cells were incubated for 15 min at room temperature after the first wash of secondary antibody, followed by two more washes with PBS. Cells were mounted on a glass slide in a drop of Fluoromount-G (SouthernBiotech) and allowed to dry overnight before imaging. Images were viewed and captured on an Olympus FluoView FV1000 confocal microscope (Olympus Canada) using FV1000 ASW software.

### Immunoprecipitation

Transfected cells growing on 10 cm dishes were washed twice in ice-cold PBS and collected by centrifugation (5 min at 1,000 × g, 4°C). We lysed the cell pellets in 300 µl lysis buffer (PBS, 0.5% sodium deoxycholate (DOC), 0.5% Triton X-100 and 1x complete, EDTA-free protease inhibitor cocktail (Roche Diagnostics, Mannheim, Germany) for 2 min on ice, followed by centrifugation for 5 min at 1,000 × g, 4°C. Lysate was removed to a fresh tube. For immunoprecipitation experiments, 100 µl cell lysate was added to 0.65 ml microfuge tubes followed by addition of 10 µl of antibody-coupled M-280 Tosyl-activated magnetic Dynabeads (Invitrogen, Carlsbad, CA). Tubes were mixed and incubated for 3 h at room temperature with constant rotation. Beads were then washed three times with 150 µl RIPA buffer (150 mM NaCl, 50 mM Tris-HCl, pH 8.0, 1% Nonidet P-40, 0.5% DOC, 0.1% SDS) with brief vortexing in-between washes, and boiled in SDS sample buffer containing 1% β -mercaptoethanol for 5 min. 1 µl of lysate was added directly into SDS sample buffer, boiled and used as a pre-IP control.

### Preparation of Antibody-coupled Magnetic Dynabeads

175 µl beads were washed twice in 1 ml PBS and resuspended in a final volume of 1 ml PBS. 80 µg DSE monoclonal antibody, or 50 µg pan-SOD1 (SOD100; Assay Designs, Ann Arbor, MI) polyclonal antibody was added to beads and incubated for 24 h at 37°C with constant rotation. Beads were then washed twice in 1 ml PBS with 0.1% BSA and incubated in 1 ml blocking buffer (0.2 M Tris-HCl, pH 8.5, 0.1% PBS) for 4 h at 37°C with constant rotation. Beads were then washed again in 1ml PBS with 0.1% BSA, resuspended in a final volume of 500 µl of PBS and stored at 4°C.

### Immunohistochemistry

We performed Immunohistochemistry for misfolded SOD1 on 5 µm thick sections of formalin fixed, paraffin embedded tissue sections using the Ventana BenchMark® XT automated staining system (Ventana, Tuscon, AZ) and developed with aminoethylcarbizole (AEC). The primary antibodies were DSE2-10E11C11 (1∶500, following microwave pre-treatment) and 3H1 (1∶50, following standard heat retrieval).

### Immunoblotting and Quantification

Boiled samples were analyzed on 4–20% acrylamide Tris-Glycine precast gels for 2 h at 125 V (Invitrogen, Carlsbad, CA). Proteins were electrophoretically transferred to PVDF membrane, blocked with 5% milk in Tris-buffered saline, 0.1% Tween-20 (TBST) for 30 min and incubated with 1 µg/ml pan-SOD1 antibody (SOD100; Assay Designs, Ann Arbor, MI) in 5% milk-TBST overnight at 4°C with constant rocking. Membranes were washed with TBST followed by 1 h incubation with donkey anti-rabbit IgG horseradish peroxidase linked whole antibody (GE Healthcare, Buckinghamshire, UK) diluted 1∶5,000 in 5% milk-TBST. Membranes were then developed with SuperSignal West Femto chemiluminescent substrate (Thermo Scientific, IL) and visualized using a VersaDoc Imager (Bio-Rad Laboratories, Hercules, CA); signal intensities were quantified using Quantity One software (Bio-Rad Laboratories, Hercules, CA). All images were acquired with no digital or biological signal saturation. In order to determine the percentage of misfolded SOD1 in each sample, quantified immunoprecipitate was compared to mIgG control, and normalized to total immunoprecipitable SOD1. The percentage of misfolded SOD1 in cell lysates is determined by the ratio of the true DSE-mAb immunoprecipitation to that of IgG2a-control, followed by normalization to total SOD1 in the lysates.

### Statistical Analysis

Without any assumption regarding the distribution underlying our small samples we applied nonparametric tests (Kruskal-Wallis and Mann-Whitney-U tests). The significance thresholds were also adjusted for multiple comparisons by the Bonferroni correction to maintain the familywise error rate and keep the alpha level at 0.05.

## Supporting Information

Figure S1 **Normal distribution of FUS and TDP43 in the cell.** (**A, B**) Untreated human neuroblastoma SH-SY5Y cells (**A**) and primary neural cells (**B**) probed for misfolded SOD1 (green) and Hoechst 33342 nuclear counter-stain (blue). Staining of the respective cells against FUS and TDP43 (top, bottom), shows completely nuclear localization of these proteins with no detectable misfolded SOD1. Scale bar, 20 µm.
(TIF)

Figure S2 **Expression of GFP in SH-SY5Y cells.** (**A**) Human neuroblastoma cells transiently expressing the exogenous GFP protein (green) do not contain misfolded SOD1, as is evident by staining with 3H1 (red). (**B**) Immunoprecipitations of SOD1 from SH-SY5Y cultures that were transfected with the GFP vector, show pull-down of total SOD1 using the pan-SOD1, SOD100 antibody, but no presence of misfolded SOD1, as is seen by lack of pull-down of both 3H1 and 10C12. (**C**) Presence of normalized % of misfolded SOD1 in eGFP transfected cultures is comparable to ev control. Error bars show s.e.m. Scale bar, 20 µm.
(TIF)

Table S1 **Clinical and demographic information on spinal cord tissues used for immunohistochemistry.** All tissues were collected at autopsy within 48 hours of patient death and used directly for IHC analysis. n/a, not available.
(TIF)
