# Supplementary figures and images for: Aberrant Localization of FUS and TDP43 Is Associated with Misfolding of SOD1 in Amyotrophic Lateral Sclerosis
Source: PLoS One. 2012 Apr 6;7(4):e35050. doi: 10.1371/journal.pone.0035050 (PMC3320864; doi:10.1371/journal.pone.0035050)

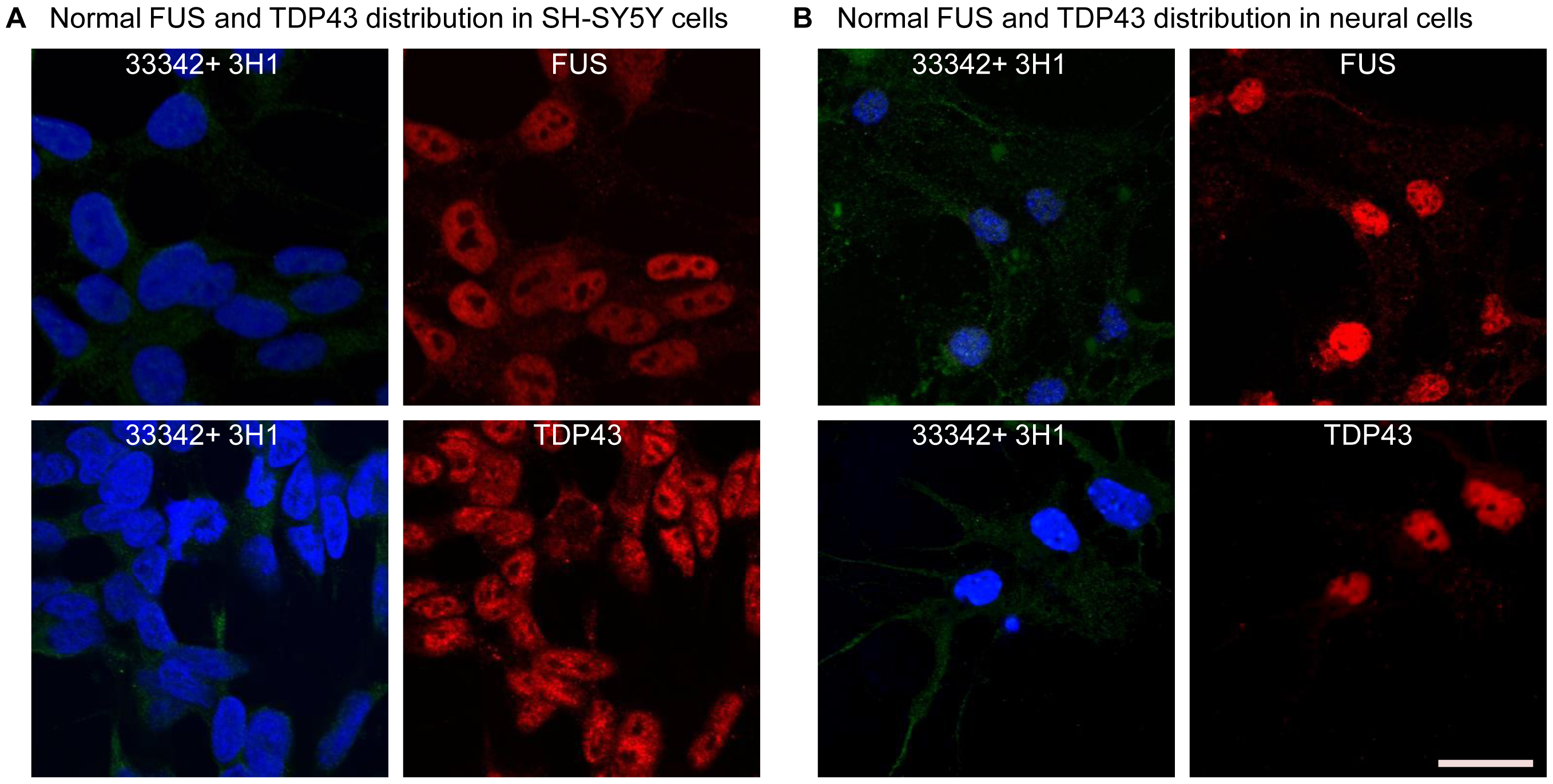

Supplement: Figure S1 — Normal distribution of FUS and TDP43 in the cell. (A, B) Untreated human neuroblastoma SH-SY5Y cells (A) and primary neural cells (B) probed for misfolded SOD1 (green) and Hoechst 33342 nuclear counter-stain (blue). Staining of the respective cells against FUS and TDP43 (top, bottom), shows completely nuclear localization of these proteins with no detectable misfolded SOD1. Scale bar, 20 µm. (TIF) [file pone.0035050.s001.tif]

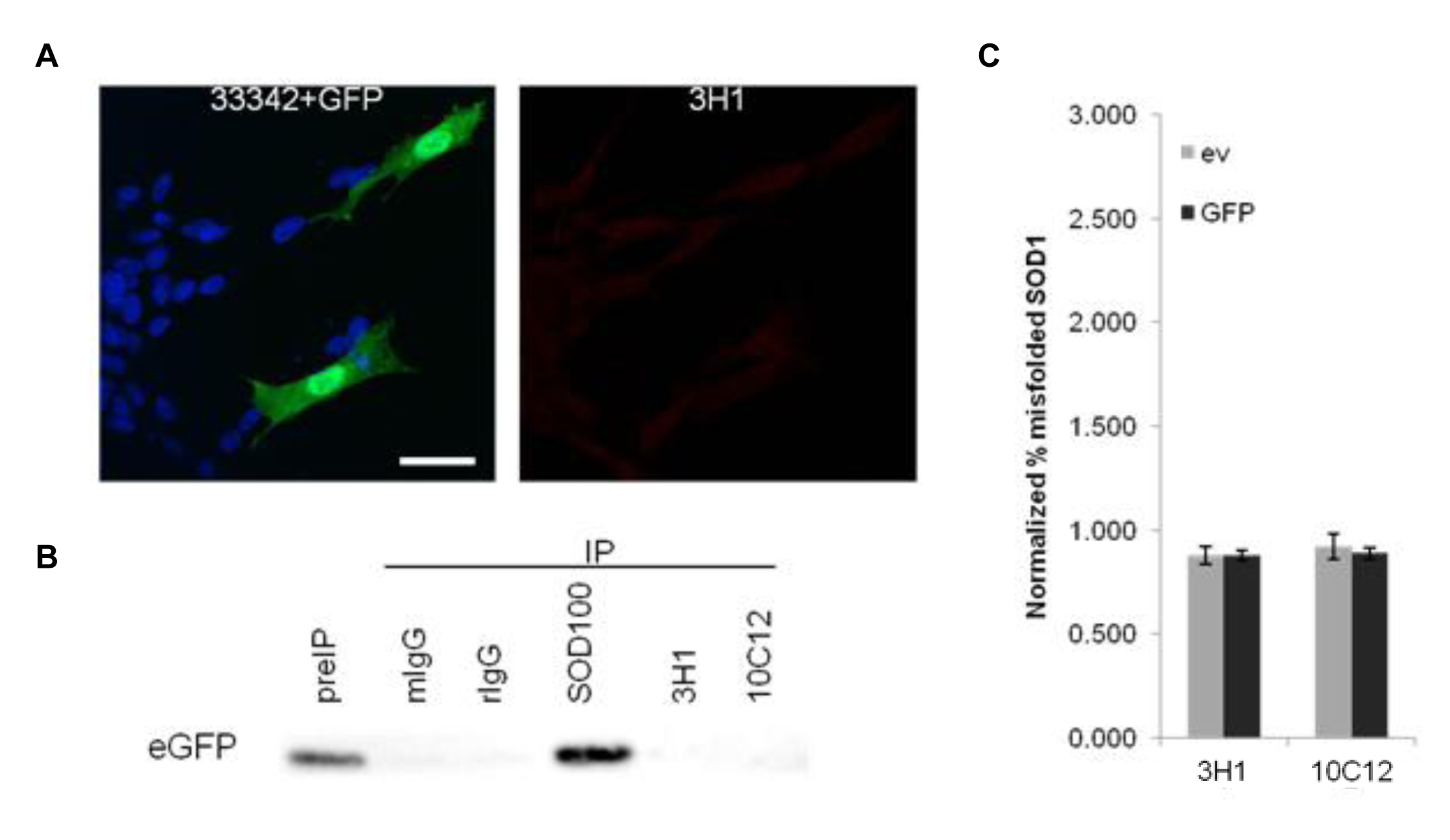

Supplement: Figure S2 — Expression of GFP in SH-SY5Y cells. (A) Human neuroblastoma cells transiently expressing the exogenous GFP protein (green) do not contain misfolded SOD1, as is evident by staining with 3H1 (red). (B) Immunoprecipitations of SOD1 from SH-SY5Y cultures that were transfected with the GFP vector, show pull-down of total SOD1 using the pan-SOD1, SOD100 antibody, but no presence of misfolded SOD1, as is seen by lack of pull-down of both 3H1 and 10C12. (C) Presence of normalized % of misfolded SOD1 in eGFP transfected cultures is comparable to ev control. Error bars show s.e.m. Scale bar, 20 µm. (TIF) [file pone.0035050.s002.tif]

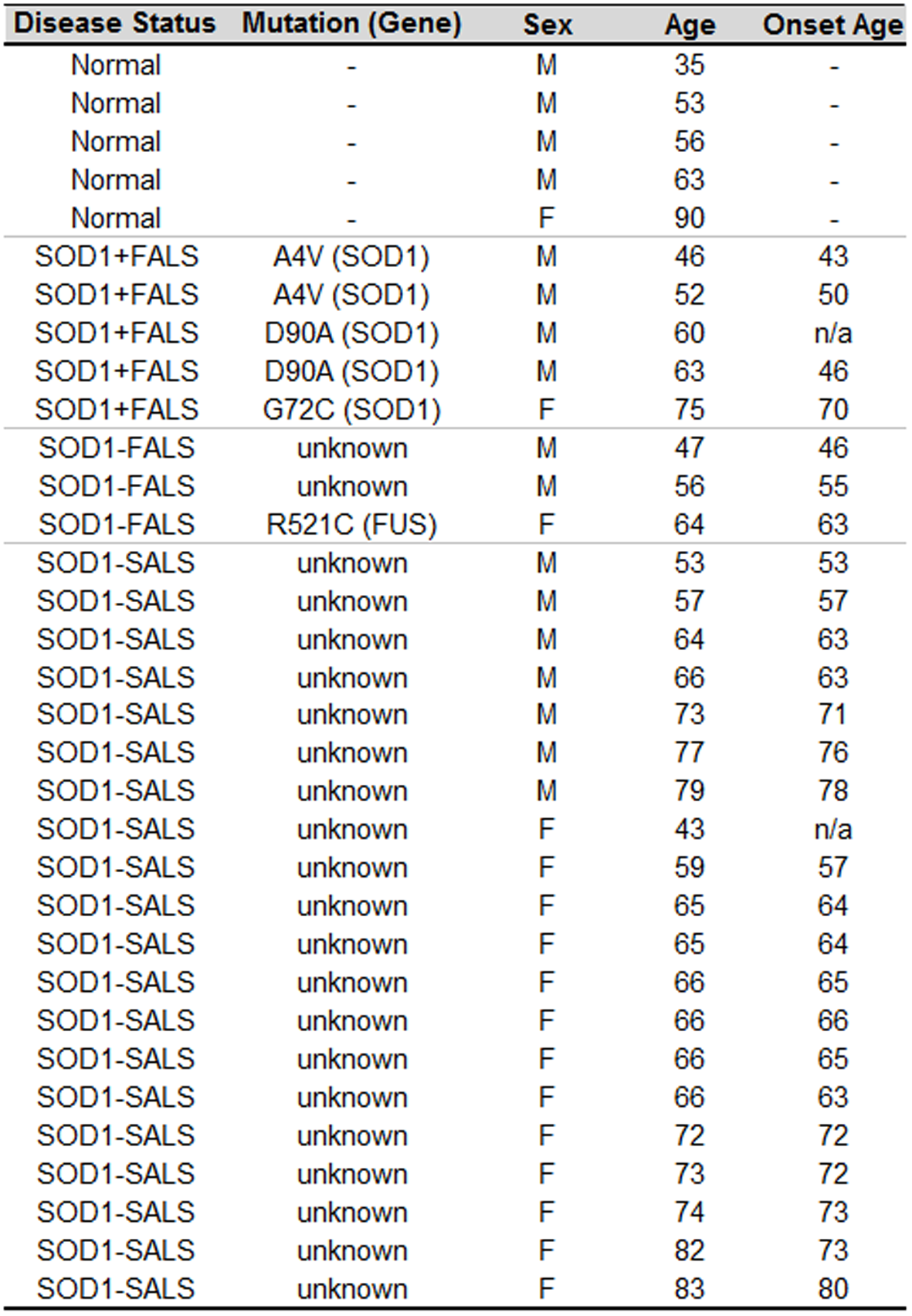

Supplement: Table S1 — Clinical and demographic information on spinal cord tissues used for immunohistochemistry. All tissues were collected at autopsy within 48 hours of patient death and used directly for IHC analysis. n/a, not available. (TIF) [file pone.0035050.s003.tif]
